# Synergistic effect of cisplatin chemotherapy combined with fractionated radiotherapy regimen in HPV-positive and HPV-negative experimental pharyngeal squamous cell carcinoma

**DOI:** 10.1038/s41598-020-58502-9

**Published:** 2020-01-31

**Authors:** Simona Kranjc Brezar, Ajda Prevc, Martina Niksic Zakelj, Andreja Brozic, Maja Cemazar, Primoz Strojan, Gregor Sersa

**Affiliations:** 1 Department of Experimental Oncology, Institute of Oncology Ljubljana, SI-1000 Ljubljana, Slovenia; 2 Faculty of Health Sciences, University of Primorska, SI-6310 Izola, Slovenia; 3 Department of Cytology and Pathology, Institute of Oncology Ljubljana, SI-1000 Ljubljana, Slovenia; 4 Department of Radiation Oncology, Institute of Oncology Ljubljana, SI-1000 Ljubljana, Slovenia; 5 Faculty of Health Sciences, University of Ljubljana, SI-1000 Ljubljana, Slovenia

**Keywords:** Oral cancer detection, Oral cancer detection

## Abstract

HPV infection renders oropharyngeal squamous cell carcinomas more radiosensitive, which results in a favorable prognosis for HPV-positive patients treated with radiation alone or with concurrent platinum-based chemotherapy. The degree of radiosensitivity in fractionated regimens has not yet been fully explored; therefore, in this study, the radiosensitivity of HPV-negative tumors (FaDu) was compared to that of HPV-positive tumors (2A3) subjected to concurrent cisplatin chemotherapy and fractionated versus isoeffective single-dose tumor irradiation in immunodeficient mice. HPV-positive tumors were approximately 5 times more radiosensitive than HPV-negative tumors, irrespective of the irradiation regimen. In both tumor models, concurrent cisplatin chemotherapy and the fractionated regimen induced significant tumor radiosensitization, with a 3- to 4-fold increase in the tumor growth delay compared to that of single-dose irradiation. Furthermore, the degree of radiosensitization induced by cisplatin chemotherapy concurrent with the fractionated irradiation regimen was much higher in HPV-positive tumors, where a synergistic antitumor effect was observed. Specifically, after combined therapy, a 26% higher survival rate was observed in mice with HPV-positive tumors than in mice with HPV-negative tumors. These data suggest that HPV-positive tumors are more radiosensitive to fractionated regimen than to single-dose irradiation with concurrent cisplatin chemotherapy acting synergistically to irradiation.

## Introduction

HPV-positive squamous cell carcinoma (SCC) of the oropharynx is an increasingly common disease and is in many ways distinct from its HPV-negative counterpart, which is caused by smoking and excessive alcohol consumption^1^. In the clinic, it was observed that HPV-positive oropharyngeal SCC responds better to treatment with radiotherapy and concurrent platinum-based chemotherapy^2,3^.

This observation was confirmed in a number of preclinical and clinical studies^2,4–10^. Furthermore, the underlying mechanisms were explored. These studies suggested that the reason for the better response of HPV-positive SCC to the treatment may be impaired DNA damage repair in HPV-infected tumor cells^5–7^. This impairment is probably caused by HPV-viral proteins, E6 and E7, which interfere with the host cell cycle to promote the reproduction of the virus^11–13^. Furthermore, the immune response to viral proteins may contribute to better treatment outcomes in HPV-positive tumors, as the presence of HPV antigens may render the tumors more immunogenic^14^.

Due to the improved response of HPV-positive oropharyngeal SCC to nonsurgical treatments, several studies evaluating different protocols of treatment de-escalation were initiated in patients with these tumors. The results of these studies showed that an approximately 20–30% deintensification of radiotherapy in combination with concurrent chemo- or immunotherapy in HPV-positive tumors is feasible and safe and leads to fewer side effects, with preservation of tumor control and survival rates^15–17^.

In our previous study of experimental tumor xenografts, we demonstrated a 20% increase in radiosensitivity in HPV-positive 2A3 tumors compared to that in HPV-negative FaDu tumors by single-dose tumor irradiation^6^. We proposed impaired DNA repair mechanisms and G_2_/M cell accumulation as possible underlying mechanisms^6^. However, the degree of radiosensitization in fractionated regimens with concurrent cisplatin treatment, a standard treatment for these tumors in the clinic, which may also add to radio-immunosensitization, has not yet been fully explored. Therefore, the aim of the present study was to compare HPV-positive tumors to HPV-negative tumors in response to concurrent cisplatin chemotherapy and fractionated irradiation in comparison to isoeffective single-dose tumor irradiation.

## Materials and Methods

### Cell lines

All cell lines were cultured at 37 °C in a 5% CO_2_ humidified atmosphere. The pharyngeal SCC cell line FaDu (ATCC®, Gaithersburg, MD) was cultured in RPMI 1460 medium (Thermo Fisher Scientific Inc., Rockford, IL). The HPV E6 and E7 viral protein-expressing cell line 2A3 (derived from the FaDu cell line, a gift from Prof. Dadachova^18^) was cultured in Dulbecco’s Minimum Essential Medium (Thermo Fisher Scientific) supplemented with 1 mg mL^−1^ G418 disulfate salt solution (Sigma-Aldrich LLC, St. Louis, MO). Both media were supplemented with 5% fetal bovine serum (Thermo Fisher Scientific), 10 mM L-glutamine (Thermo Fisher Scientific), 100 U/mL penicillin (Grünenthal, Aachen, Germany) and 50 mg/mL gentamicin (KRKA, Novo Mesto, Slovenia). The methods used to determine the presence and expression of the HPV16 E7 gene in the 2A3 cell line and the corresponding results are described in Prevc *et al*. 2018, Supplementary Materials^6^.

### Cell cycle analysis

**Figure 1:**
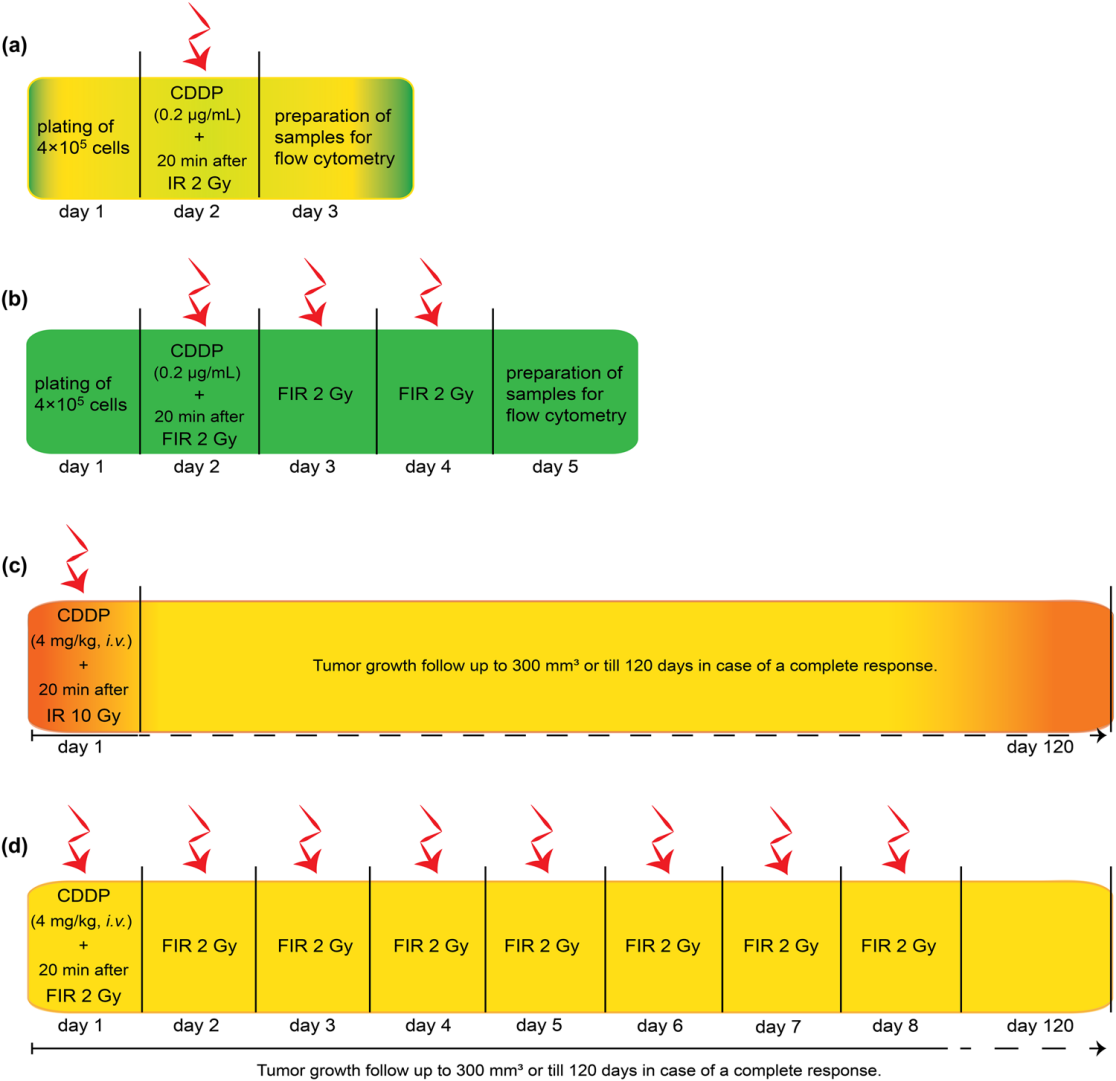
Scheme of the treatment of HPV-negative (FaDu) and HPV-positive (2A3) cells and tumors with concurrent cisplatin (CDDP) and a single-dose or fractionated irradiation regimen. (**a**) Time course of cell treatment with concurrent CDDP and a single-dose irradiation (2 Gy, IR) on day 2 for flow cytometry analyses. (**b**) Time course of cell treatment with concurrent CDDP fractionated irradiation regimen (3 × 2 Gy, FIR) for flow cytometry analyses. (**c**) Time course of tumor treatment with concurrent CDDP and IR (10 Gy) (**d**) Time course of tumor treatment with concurrent CDDP and FIR (8 × 2 Gy). CDDP was injected intravenously 20 min prior to single-dose or fractionated irradiation regimen on the first day only.

The cells were plated on Petri dishes at a density of 4 × 10^5^ and incubated for 24 h. For the combined treatment, 0.2 µg/mL cisplatin was added to the cell medium 20 min prior to irradiation (Fig. 1a). The cells were then irradiated with 2 Gy (single dose) or three times 2 Gy (fractionated irradiation, one fraction per day) at a dose rate of 1.8 Gy min^−1^ using a Gulmay 225 X-ray system (Gulmay Medical Ltd.) with 0.55 mm copper and 1.8 mm aluminum filtering. Twenty-four hours after the treatment (Fig. 1a,b), the cells were trypsinized and prepared for flow cytometry DNA analysis according to modified Otto’s method^19^. Briefly, the cells were resuspended in 0.2 M citric acid (Farmalabor Srl, Canosa di Puglia, Italy) and 0.5% Tween-20 (VWR Chemicals, France) for 20 min, centrifuged for 5 min at 470 g (Heraeus MULTIFUGE 1S-R, Kendro Laboratory Products GmbH, Langenseebold, Germany) and fixed in 3 ml of 70% alcohol at 4 °C. Twenty-four hours later, the cells were centrifuged (470 g, 5 min), washed once with PBS, again centrifuged for 5 min at 470 g, resuspended in 50 µL of RNase A (100 µg/mL, Qiagen GmbH, Hilden, Germany) and stained with the DNA binding dye propidium iodide (50 µg/mL, Sigma Aldrich, Buchs Switzerland). Then, cell cycle analysis of the samples was performed using a FACSCanto II flow cytometer (BD Biosciences). DNA histograms of the number of cells against the observed fluorescence intensity were obtained. The resulting cell cycles were analyzed with ModFit LT software (Verity Software House). For each sample, the distribution of cells in the G_0_/G_1_, G_2_/M and S phases was determined.

### *In vivo* study

Female SCID mice (6–8 weeks old, C. B-17/IcrHsd-Prkdcscid; Envigo, Italy) were maintained on a 12 h light–dark schedule under specific pathogen-free conditions at constant room temperature and humidity. Food and water were provided *ad libitum*. For induction of subcutaneous tumors, a 100 μL suspension of 2 × 10^6^ FaDu or 5 × 10^6^ 2A3 cells, prepared from cell cultures *in vitro*, was injected subcutaneously into the shaved backs of the mice. At a 40 mm3 tumor volume, the mice were randomly divided into groups of at least 8.

Treatment protocols were approved by the Ministry of Agriculture, Forestry and Food of the Republic of Slovenia (permission No. 34401-1/2015/16) based on the approval of the National Ethics Committee for Experiments on Laboratory Animals. Animal experiments were carried out in accordance with the UK Animals (Scientific Procedures) Act, 1986, and in accordance with the guidelines for animal experiments of the EU Directive 2010/63/EU.

For combined treatment with cisplatin, cisplatin (4 mg/kg, 100 μL, Cisplatina Kabi; Fresenius Kabi AG, Bad Homburg, Germany) was injected intravenously, with the volume depending on the weight of the mice. Twenty minutes after the cisplatin injection, we performed either single-dose irradiation or the first fraction of the fractionated regimen (Fig. 1c,d). For local irradiation, mice were placed in Pb carriers, and the tumors were irradiated. For uniform dose distribution throughout the tumor volume, two opposite sides of the tumor were irradiated with 50% of the total dose (dose rate 1.92 Gy min^−1^) using a Gulmay 225 X-ray system (Gulmay Medical) with 0.55 mm copper and 1.8 mm aluminum filtering. Mice were irradiated either by a single dose of 10 Gy or by 8 fractions of 2 Gy (one fraction per day in 8 consecutive days) (Fig. 1c,d). The number of fractions was selected as a 2 Gy fraction regimen, isoeffective to a 10 Gy single dose, calculated by the following Eq. (1),

1 $${{\rm{D}}}_{1}/{{\rm{D}}}_{2}=(\alpha /{\rm{\beta }}+{{\rm{d}}}_{2})/(\alpha /{\rm{\beta }}+{{\rm{d}}}_{1})$$

where D_1_ was the total single-dose irradiation, d_1_ was the total fraction of single-dose irradiation, α/β was estimated to be 10 Gy for head and neck SCC tumors, and d_2_ was the fraction size, set to 2 Gy^20^.

Tumor growth was monitored three times a week by measuring the tumor diameters using a Vernier caliper until the tumor volume reached 300 mm3 (data used for Kaplan-Meier survival curves). Tumor volume was calculated using the Eq. (2),

2 $$V=(\pi \times {\rm{a}}\times {\rm{b}}\times {\rm{c}})/6$$

where a, b and c are three perpendicular dimensions of the tumor. Tumor growth delay was calculated as the difference between the tumor doubling time of the treated groups and the doubling time of the pertinent control group. In addition, general animal well-being was determined by monitoring the animal weight and normal tissue damage by the skin reaction in the irradiated field around the tumor (5 mm), three times per week.

### Statistical analysis

Statistical analysis and graphical representation were performed using SigmaPlot Software (Systat Software, UK). Data were tested for normality of distribution using Shapiro-Wilk test. The arithmetic mean (AM) and standard error of the mean (SE) were calculated. Statistically significant differences between experimental groups were determined using one-way analysis of variance (one-way ANOVA) followed by a Holm–Sidak test. The difference between experimental groups was considered significant if the p-value was <0.05. In addition, the mode of action (additivity, synergism, and antagonism) of treatments with independent mechanisms was evaluated by the method developed by Spector *et al*.^21^. Survival estimates were obtained by the method of Kaplan-Meier and compared by the log-rank test^22,23^.

## Results

### Single treatments

**Figure 2:**
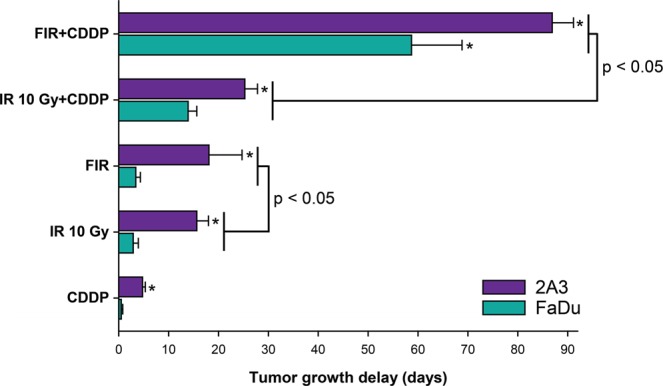
Tumor growth delay of HPV-negative (FaDu) and HPV-positive (2A3) tumors after concurrent cisplatin (CDDP) treatment combined with single (IR, 10 Gy) or fractionated irradiation (FIR, 8 × 2 Gy). Data represent AM ± SE, n = 8–12. *p < 0.05 between tumor models.

In this study, we used FaDu tumors as an HPV-negative tumor model and 2A3 tumors as an HPV-positive tumor model. Tumors were irradiated with two isoeffective regimens, i.e., 10 Gy single-dose irradiation or a fractionated regimen (8 × 2 Gy). Indeed, the tumor growth delays after 10 Gy single-dose irradiation and fractionated irradiation were similar in both tumor models. The tumor growth delay in FaDu tumors was 2.9 ± 1.0 days after single-dose irradiation and 3.4 ± 0.9 days after fractionated irradiation, p = 0.31; in 2A3 tumors, it was 15.6 ± 2.3 days after single-dose irradiation and 18.1 ± 6.6 days after fractionated irradiation, p < 0.05. However, 2A3 tumors were 5.3 times more radiosensitive to 10 Gy single-dose irradiation (p < 0.001) and 5.4 times more sensitive to fractionated irradiation (p < 0.05). In addition, 2A3 tumors were 2.6 times more sensitive to cisplatin treatment alone (p < 0.05) than FaDu tumors (Fig. 2).

### Concurrent cisplatin treatment and irradiation

When tumors were irradiated in combination with concurrent single cisplatin administration 20 min before irradiation, significant radiosensitization was observed in both tumor models. The tumor growth delay after concurrent cisplatin with single-dose irradiation was 13.9 ± 1.7 days (p < 0.05 to single-dose irradiation alone) in FaDu tumors, while in 2A3 tumors, it was 25.3 ± 2.6 days (p < 0.05 to single-dose irradiation alone) (Fig. 2). Thus, 2A3 tumors were more radiosensitive in combined treatment than FaDu tumors, resulting in a 1.8 times longer tumor growth delay after concurrent cisplatin chemotherapy with single-dose irradiation.

Furthermore, radiosensitization was significantly greater when cisplatin was combined with a fractionated regimen than when cisplatin was combined with single-dose irradiation (p < 0.05) (Fig. 2). In fact, the combined therapy had a synergistic effect in both tumor models, calculated according to Spector’s formula. Specifically, the tumor growth delay was 58.7 ± 10.1 days in FaDu tumors (p < 0.05 to fractionated irradiation) and 86.9 ± 4.3 days in 2A3 tumors (p < 0.05 to fractionated irradiation) (Fig. 2). Again, the effect was more pronounced in HPV-positive 2A3 tumors than in FaDu tumors, with a 1.5 times longer tumor growth delay after concurrent cisplatin chemotherapy with the fractionated regimen (p < 0.05).

**Figure 3:**
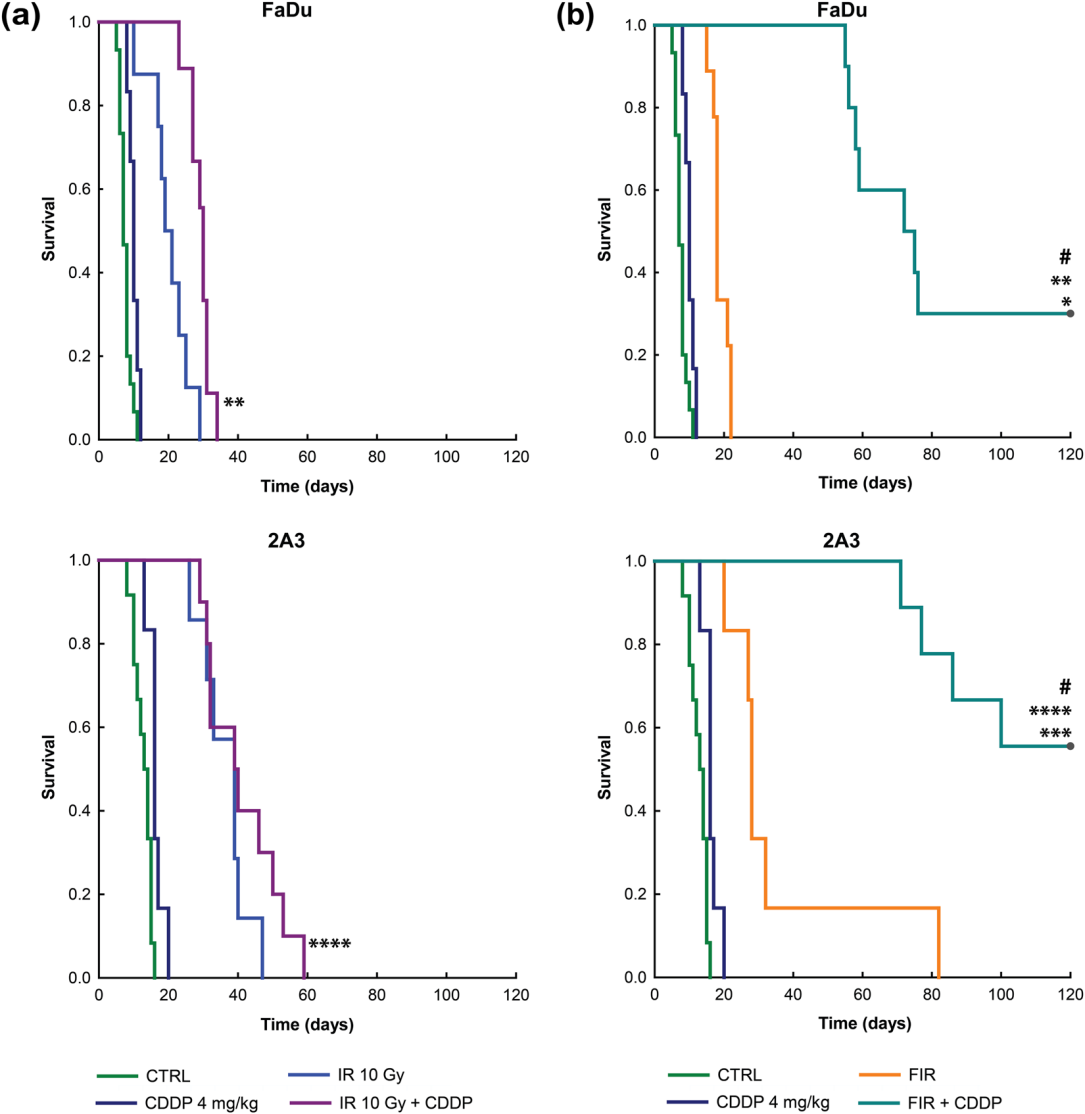
Kaplan-Meier survival curves of the mice with HPV-negative (FaDu) or HPV-positive (2A3) tumors after concurrent treatment with *i.v*. injection of cisplatin (CDDP) and single (IR) (**a**) or fractionated irradiation (FIR) (**b**). *p < 0.05 between treatments for FIR *vs* FIR + CDDP in FaDu tumors; **p < 0.05 between treatments for IR 10 Gy + CDDP *vs* FIR + CDDP in FaDu tumors; ***p < 0.05 between treatments for FIR *vs* FIR + CDDP in 2A3 tumors; ****p < 0.05 between treatments for IR 10 Gy + CDDP *vs* FIR + CDDP in 2A3 tumors; ^#^p < 0.05 between treatments for FIR + CDDP in FaDu *vs* FIR + CDDP in 2A3 tumors; n = 8–12 mice. CTRL, the control group.

Furthermore, concurrent cisplatin treatment combined with the fractionated regimen also resulted in superior survival rates in both tumor models, while there were no cures after the combination of single-dose irradiation and cisplatin chemotherapy. A 26% difference in survival rates was observed between HPV-positive 2A3 tumors and HPV-negative FaDu tumors in favor of the former (56% vs. 30%, p < 0.05) (Fig. 3).

### Toxicity

Single treatments alone, as well as combination of cisplatin treatment with single-dose or fractionated irradiation, did not evoke any systemic toxicity. Maximal animal weight loss in the group of mice treated with cisplatin and single-dose irradiation was 5% in the first 14 days after treatment completion (data not shown). Skin reactions were observed only in the group of mice treated with cisplatin combined with single-dose irradiation. Thirty percent of animals presented skin reactions, which manifested as edema and mild erythema that completely resolved 25 days after the treatment.

### Cell cycle distribution

**Figure 4:**
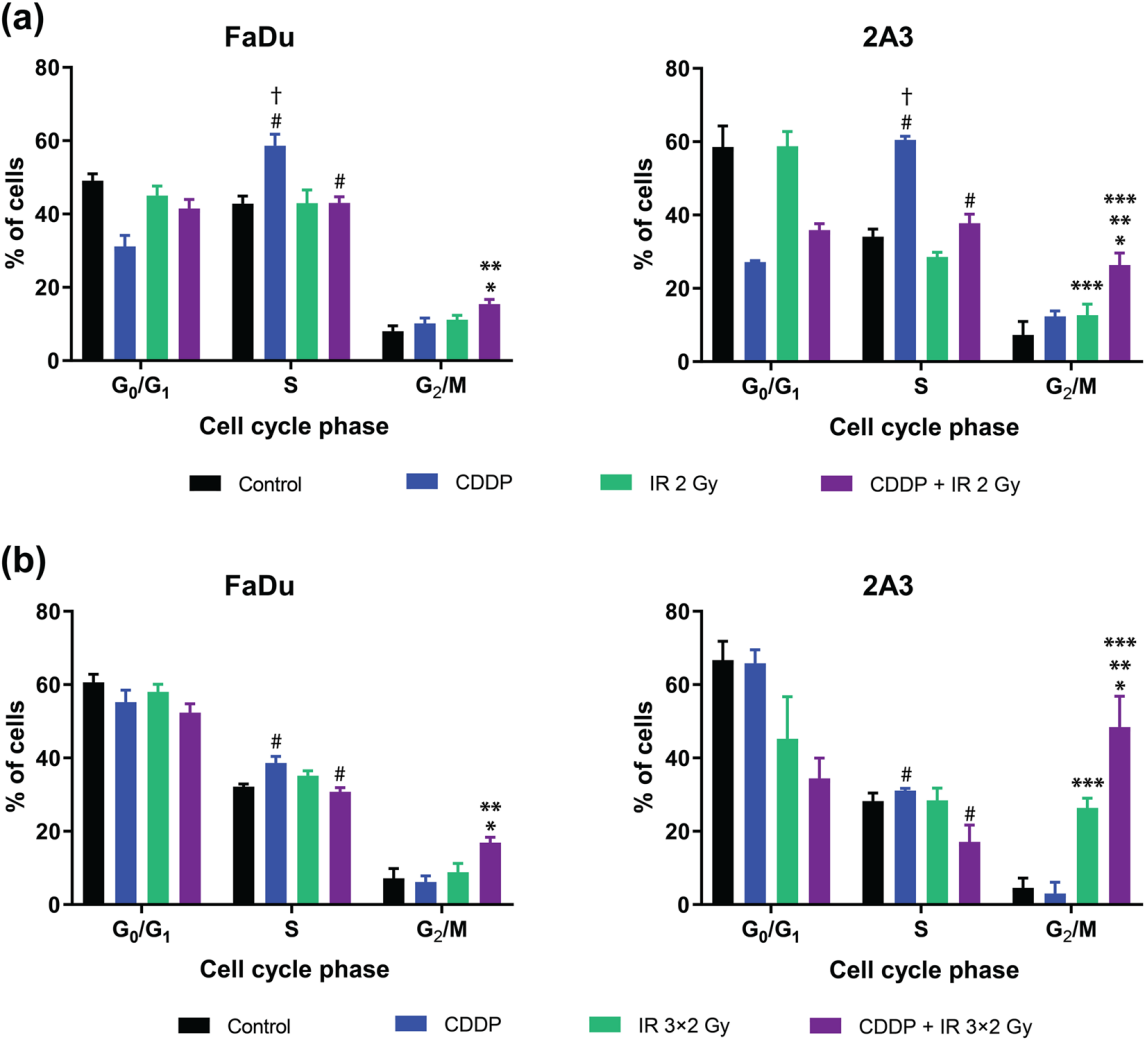
Cell cycle redistribution of HPV-negative (FaDu) and HPV-positive (2A3) cells after cisplatin (CDDP) treatment combined with single-dose (IR 2 Gy) (**a**) or fractionated irradiation (IR 3 × 2 Gy) (**b**). In the single-dose regimen, cells were analyzed 24 h after irradiation, and fractionated regimen cells were analyzed 24 h after the last irradiation. Data were obtained from three independent experiments with 5,000 events measured in each sample (mean ± standard error). *p < 0.05 to other experimental groups in G_2_/M phase; **p < 0.05 between tumor models; ***p < 0.05 between single and fractionated irradiation alone or combined with cisplatin in 2A3 cells in G_2_/M phase; ^†^<0.05 to other experimental groups in the S phase ^#^p < 0.05 between cisplatin and irradiation groups in the single *vs* fractionated regimen in the corresponding tumor model.

To explore the mechanism of radiosensitization, cell cycle redistribution was measured 24 h after cisplatin treatment combined with single-dose irradiation (2 Gy) or 24 h after the third fraction of irradiation in the fractionated regimen combined with cisplatin treatment *in vitro*. In both schedules, cisplatin was administered only once, i.e., 20 min before the first 2 Gy irradiation. The cell cycle analysis in HPV-negative and HPV-positive cells demonstrated primarily S phase arrest 24 h after cisplatin treatment alone; compared to the irradiation and combined treatment a significantly higher proportion of cells was obtained in S phase (p < 0.05). The combined cisplatin treatment with single-dose as well fractionated irradiation resulted in G_2_/M phase arrest; compared to the control groups (treatment with cisplatin or irradiation alone; p < 0.05), a significantly higher proportion of cells was obtained in G_2_/M phase. This G_2_/M arrest was significantly more evident in HPV-positive 2A3 cells than in HPV-negative FaDu cells (p < 0.05) and was 72 h later even more pronounced. In addition, combined treatment with the fractionated irradiation regimen and cisplatin resulted in a higher proportion of 2A3 cells in G_2_/M phase than treatment with cisplatin and single-dose irradiation, which may contribute to increased sensitivity *in vivo* (Fig. 4). In both tumor cell lines, the percentage of the cells in S phase significantly decreased after fractionated irradiation (p < 0.05) compared to that observed after single-dose tumor irradiation (Fig. 4).

## Discussion

Our previous study demonstrated that HPV-positive pharyngeal tumors were 30% more sensitive to single-dose irradiation and 20% more sensitive to concurrent cisplatin therapy than HPV-negative tumors. The current study confirms the previous findings and extends them to a clinically relevant fractionated regimen. These results suggest that HPV-positive tumors respond better to fractionated radiotherapy with or without concurrent cisplatin chemotherapy than HPV-negative tumors. A 3- to 4-fold increase in tumor growth delay was observed after fractionated irradiation compared to single-dose irradiation, together with superior survival when combined with concurrent cisplatin therapy. A difference of 26% was found between survival rates recorded in HPV-positive and HPV-negative tumors (56% *vs*. 30%, p < 0.05). These data indicate that HPV-positive tumors are more sensitive to fractionated irradiation than to single-dose irradiation with concurrent cisplatin treatment. A higher sensitivity of HPV-positive tumors to cisplatin treatment alone and cell cycle redistribution during fractionated irradiation even after the first fraction, i.e., higher accumulation of HPV-positive cells than HPV-negative cells in G_2_/M phase, which is most sensitive to irradiation, could be one of the underlying mechanisms for the increased efficacy of this combined treatment.

Overall, patients with HPV-positive oropharyngeal SCC tumors respond better to radiotherapy than those with HPV-negative tumors and have a better prognosis^2,24,25^. The mechanisms of increased radiosensitivity observed in HPV-positive cells have been the subject of many studies^4,5,7,8,26–30^. It has been suggested that this effect is due to impaired DNA repair mechanisms, cell cycle dysregulation and increased levels of reactive oxygen species, caused mainly by HPV-related proteins E6 and E7. In fact, E6 protein binds to the p53 protein, and E7 binds to the Retinoblastoma (Rb) protein, which both mediate proteasomal degradation of targeted proteins and consequently abrogate apoptosis and senescence in host cells or arrest cells in G_2_/M phase, which is the most vulnerable to irradiation^4,8,31–33^. Furthermore, irradiation of HPV-positive cells induces fast progression of cells through S phase with subsequent arrest in G_2_/M phase^4,6,8^. The cell accumulation in this phase of the cycle correlates with increased levels of apoptosis and DNA double-strand breaks and decreased levels of mitosis *in vitro*^4,6–8^. Consequently, this may lead, together with other mechanisms, to higher radiosensitivity (up to 30%) of HPV-positive tumors *in vivo*, as observed in our previous study on single-dose irradiation^6^. In the current study, we determined an increased proportion of HPV-positive cells in G_2_/M phase after a single-dose or fractionated irradiation regimen *in vitro*. Additionally, a better response of HPV-positive tumors *in vivo* to both single-dose (up to 5.4-times prolonged tumor growth delay) and fractionated irradiation regimens (up to 5.3-times prolonged tumor growth delay) was recorded compared to that of their HPV-negative counterparts, thus confirming our previous results and expending them to the fractionated regimen. These results are in line with recent findings in clinical studies where treatment of a reduced intensity in HPV-positive oropharyngeal tumors was found to be equally effective as standard treatment regimens^15,17^. The results of these studies showed that a 20–30% decrease in the total dose of fractionated irradiation (from standard 70 Gy to 56 Gy or 50 Gy) for HPV-positive tumors is safe and feasible, with local control and survival remaining as high as those observed after the standard irradiation dose^16,17^.

In published studies, different HPV-positive cell lines have shown considerable variations in sensitivity not only to irradiation^7,26,27^ but also to cisplatin compared to HPV-negative cells^8,34^, and the same was observed in the combined treatment setting. Thus, the degree of radiopotentiation can vary; therefore, one of limitations of our study is the use of only one tumor model. Further studies employing other tumor models are warranted to confirm the radiopotentiating effect obtained by cisplatin in combination with fractionated irradiation. In clinical practice, cisplatin adds a benefit to radiotherapy, with significant improvement of overall survival at five years in head and neck SCC patients^35,36^. It is known that cisplatin treatment increases the duration of S phase and arrests cells in the G_2_/M phase^8,34,37–39^, which could contribute to radiosensitization. In the present study we share similar observations. Furthermore, radiation-induced arrest in the G_2_/M phase occurred later and was more sustained in HPV-positive than HPV-negative cells^4^. Our data demonstrate a significant increase in G_2_/M arrest after concurrent cisplatin treatment combined with a single-dose or fractionated irradiation regimen, which is more pronounced (up to 2.9-fold) in the HPV-positive tumor model than in the HPV-negative model. The obtained results differ slightly from the previous ones^6^ and the reason might be the lower dose of irradiation (2 Gy compared to 5 Gy) and a slightly higher dose of cisplatin (0.2 μg/ml compared to 0.1 μg/ml). A higher dose of irradiation was found to induce higher G_2_/M arrest in our previous study^6^ and also shown in other studies^4,7,8^.

Interestingly, both tumor models responded much better when cisplatin treatment was combined with fractionated irradiation than with single-dose irradiation; in the former case, the effect was synergistic. In fact, the tumor growth delay after combined treatment with cisplatin and fractionated irradiation was improved up to 4 times in both tumor models compared to that of the single-dose radiation modality. The survival difference between the two tumor models when treated with fractionated irradiation was 26% in favor of HPV-positive tumors, which had a 1.5 times longer tumor growth delay.

One of the possible mechanisms responsible for the synergistic effects of cisplatin treatment concurrent with the fractionated regimen is the involvement of immunomodulation by the combined treatment. Immunomodulatory effects of radiation and cisplatin treatment have been previously confirmed in preclinical and clinical studies^40–47^. Radiation can induce an immunogenic death of tumor cells, which then serve as a source of tumor antigens. Additionally, cisplatin-induced major histocompatibility complex I expression, improved recruitment and proliferation of immune effector cells, upregulation of lytic activity of cytotoxic effector cells and downregulation of the immunosuppressive tumor microenvironment have been shown to add to the antitumor response^42^. Thus, an important shortcoming of our study remains the inability to evaluate the contribution of the immune response to the antitumor effects, as the tumors were growing on immunodeficient mice. Indeed, radiation therapy with concurrent cisplatin resulted in a partial response of tumors in the absence of an immune response (immunodeficient B6129S7-Rag1^un1Mom^/J mice), while 50% of the complete response was obtained in immunocompetent animals (C57Bl/6 mice)^26^. Furthermore, boosting the immune response with the modulated adenoviral vaccine that targets HPV oncogenes E6 and E7 in combined chemoradiotherapy treatment cured 90% of HPV-positive tumors in immunocompetent animals but none in immunodeficient animals^26,48,49^. Additional research revealed that irradiation of HPV-positive cancer cells induces the reduction of CD47 (cluster of differentiation 47) expression, which stimulates phagocytosis via dendritic cells and results in immune-mediated cell death of HPV-positive cancer^50^. Additionally, selectively decreased CD47 expression induced by radiation of HPV-positive tumor cells in combination with cisplatin treatment efficiently improved immune-mediated tumor clearance *in vivo* in immunocompetent but not immunodeficient Rag1 mice^50^. However, the chosen treatment regimen for HPV-positive and HPV-negative tumors in immunodeficient animals in these studies^26,50^, i.e., concurrent cisplatin treatment and 8 Gy irradiation given once a week for three weeks, was less effective than ours, resulting only in a tumor growth delay and no survivors. Similar results were obtained in our study with single-dose irradiation, with or without concurrent cisplatin therapy, which resulted in a prolonged tumor growth delay without surviving animals. However, cisplatin treatment before the first fraction of irradiation and fractionated irradiation (in total 8 × 2 Gy) acted synergistically, indicating the role of irradiation as an inducer of immunogenic cell death. Previous studies have shown that low dose irradiation in itself has an effect on macrophages thereby supporting T cell responses^51^. In addition, oncoproteins E6 and E7 make HPV-positive tumors highly recognizable to the immune system, thus inducing HPV-specific T cell response, which favors tumor control^52^. However, in a proportion of patients with a HPV-positive tumor immune response to HPV is weak or even absent and subsequently has a worse outcome upon standard therapy than those a HPV-specific T cell response^52^. Therefore, we can only speculate that although immunodeficient SCID animals were used, the innate immune response was elicited, mediated mainly by natural killer cells, macrophages, and granulocytes that synergized with cisplatin therapy. Overall, local irradiation can act synergistically with other therapies, but the antitumor efficacy of combined treatment is determined by a fraction dose of the irradiation.

## Conclusion

In conclusion, we confirmed that the response of HPV-positive pharyngeal SCC is better than the response of HPV-negative tumors after fractionated irradiation. Furthermore, radiosensitization by concurrent cisplatin treatment at isoeffective irradiation doses was still present in both tumor models but was more pronounced after fractionation than after single-dose irradiation. Namely, the combined treatment acted synergistically, resulting in a 56% survival rate after the fractionated regimen in mice bearing HPV-positive tumors and only 30% in mice bearing HPV-negative tumors. The fractionated regimen increased the accumulation of cells in G_2_/M phase, which could be one of the underlying mechanisms of the improved response of HPV-positive tumor cells to chemoradiation.

## Data Availability

All data generated or analysed during this study are included in this published article.
